# Cell Biology of Viral Infections

**DOI:** 10.3390/cells9112431

**Published:** 2020-11-07

**Authors:** Pierre-Yves Lozach

**Affiliations:** CellNetworks–Cluster of Excellence and Center for Integrative Infectious Diseases Research (CIID), Virology, University Hospital Heidelberg, 69120 Heidelberg, Germany; pierre-yves.lozach@med.uni-heidelberg.de

**Keywords:** 3D cell culture, cell death, host-virus interactions, inclusion bodies, intracellular trafficking, organoid, pluripotent stem cells, signaling pathways, cell stress responses, virus

## Abstract

Viruses exhibit an elegant simplicity, as they are so basic, but so frightening. Although only a few are life threatening, they have substantial implications for human health and the economy, as exemplified by the ongoing coronavirus pandemic. Viruses are rather small infectious agents found in all types of life forms, from animals and plants to prokaryotes and archaebacteria. They are obligate intracellular parasites, and as such, subvert many molecular and cellular processes of the host cell to ensure their own replication, amplification, and subsequent spread. This special issue addresses the cell biology of viral infections based on a collection of original research articles, communications, opinions, and reviews on various aspects of virus-host cell interactions. Together, these articles not only provide a glance into the latest research on the cell biology of viral infections, but also include novel technological developments.

Viruses are particles made up of nucleic acids and proteins, and sometimes, of lipids and glycans. Viral particles differ considerably from one isolate to another in terms of their structural, molecular, and genomic organization. Their sizes range from a few tens of nanometers to micrometers, and viral genomes can encode anywhere from a few genes to several hundreds. As obligate intracellular parasites, viruses exploit host cells to replicate, amplify, and subsequently spread from cell to cell and from host to host. Thousands of viruses have been sequenced thus far, but only a few are known to cause fatal diseases. However, viral infections have a colossal impact on public health, agricultural productivity, and the economy. International trade, urbanization, deforestation, and climate change, and human activity, in brief, are all factors that promote not only the emergence and re-emergence of viruses, but also their global spread. Recent illustrations are the Zika pandemic and the lasting problem created by the infamous coronavirus SARS-CoV-2 worldwide. Understanding the infection process at the molecular and cellular level is an obvious prerequisite for the development of diagnostic tools, preventive approaches, and therapeutic strategies against viruses.

Research on the cell biology of viral infections remains limited to a relatively small number of viruses, thus posing a serious challenge in our preparedness against future emerging viral infectious diseases. However, many breakthroughs in the field have occurred over the past decades, shedding light on the cellular life cycles of important pathogens such as human immunodeficiency (HIV) and hepatitis viruses. On the other hand, many of these investigations have led to major advances in molecular and cellular biology. To cite only a few, exciting discoveries include protein G of the vesicular stomatitis virus, which contributed to defining the exocytotic machinery, the many viruses employed as functional cargo to decipher endocytic pathways, viral fusion proteins, and developmental biology, and the retroviral reverse transcriptase, currently used in various biotechnology applications. The list is not limited to cell biology and can be easily expanded to fields as diverse as immunology, neurobiology, and molecular medicine. Viruses somehow represent interesting molecular and cellular bridges between all these scientific disciplines. This is exactly what this special issue aims to illustrate, bringing together original results and discussions on various aspects of viral infection cell biology and their implications for other topics in biology.

Infection starts when viruses attach to the host cell surface, which invariably implies binding to one or more cellular receptors, including proteins, carbohydrates, and lipids. The expression of these molecules is often tissue-specific. Although other factors and processes contribute to the infection program, the identification of receptors and coreceptors helps to define the virus tropism, and sometimes, to explain the virus-induced pathogenesis. Wielgat and colleagues discuss a possible link among coronaviruses, sialic acids as attachment factors, immune escape, and disease severity [1]. Ideally, preventing infection requires approaches targeting early virus–host cell interactions, before the release of the viral genome into the cytosol. In this sense, Luteijn et al. identified a peptide with broad-spectrum antiviral activity that targets phosphatidylserine in the viral particle envelope and prevents infection by poxviruses, HIV, hepatitis B virus, and Rift Valley fever virus [2]. This finding is consistent with the fact that viruses often use phosphatidylserine and apoptotic mimicry as a mechanism of entry into host cells [3].

After attachment to the cell surface and prior to viral replication, viruses must gain access to the cytosol, and most viruses are sorted into the endocytic machinery [4]. To release their genomic material into the cytosol, enveloped viruses fuse their membrane with that of endosomal vesicles, and nonenveloped viruses usually inflict damage in the endosomal membrane. Daussy and colleagues reviewed the different strategies by which nonenveloped viruses enter the cytosol and provided the latest knowledge on how these viruses control and prevent cellular stress responses following membrane injuries such as inflammation and autophagy [5]. After delivery into the cytosol, the virus journey is not necessarily over, as they very often must reach a specific subcellular location to initiate viral replication, a point illustrated by Grikscheit et al. [6]. By combining live cell microscopy and computer-based image analysis, the researchers provided evidence that the Ebola virus (EBOV) finely regulates actin polymerization to ensure the directed long-distance transport of its nucleocapsid.

After penetration into the cytosol, viral replication usually takes place in viral factories wherein nucleic acids and specific viral and cellular proteins accumulate. Two studies by the Hoenen laboratory reported that EBOV recruits carbamoyl-phosphate synthetase 2, aspartate transcarbamylase, dihydroorotase (CAD) and the nuclear RNA export factor NXF1 within inclusion bodies in infected cells [7,8]. CAD facilitates the replication and transcription of the EBOV genome, while NXF1 contributes to the expression of EBOV proteins. Van Huizen and McInerney reviewed how Old World alphaviruses manipulate the phosphatidylinositol-3-kinase (PI3K)-AKT signaling pathway and discuss the benefit for viral replication [9]. Of note, viral factories can be either devoid of any lipidic layer, such as Negri bodies, or delimited by cellular membranes [10]. In the case of hantaviruses, Davies et al. established that the Tula virus induces a dramatic reorganization of the Golgi to replicate [11]. Once viral replication begins, cells are infected.

The fight between host cell defenses and viruses is permanent at each stage of the viral life cycle. The work by the Grandvaux team points out the existence of a noncanonical pathway leading to an antiviral, immunoregulatory response [12]. The data support the view that the two factors STAT2 and IRF9 regulate distinct pools of genes with functions related to antiviral and immunoregulatory responses when the levels of both interferon β and tumor necrosis factor are elevated. Another example is that bovine herpesvirus 1 infection results in the proteasomal degradation of the transporter associated with antigen processing (TAP), a key player in major histocompatibility complex class I (MHC-I)-restricted antigen presentation. Wachalska et al. found that the process depends on the host cell factor p97, which is involved in the complex ER-associated degradation pathway [13]. Saulle and colleagues observed that ERAP2/Iso3 expression is induced by infection with different viruses, including SARS-CoV-2 [14]. ERAP2/Iso3 is an alternatively spliced isoform of rs2248374-G ERAP2 that, unlike the full transcript, is incapable of trimming peptides to be loaded onto MHC-I molecules. These results suggest that viruses trigger ERAP2/Iso3 expression to escape the adaptive immune response, which could contribute to the severity of virus-induced disease. In summary, cells have acquired many molecular weapons to control viral infections, but viruses have developed equally as many strategies to evade cell defenses.

To infect and replicate in host cells, viruses display not only the capacity to escape immune responses, but also to control cellular stress responses such as degradative pathways, autophagy, and cell death. Vector-borne viruses typically persist in their vector, but not in other hosts [15,16]. In this regard, much more work is needed to improve our understanding of the vector-borne virus life cycle and transmission to humans and other vertebrates.

Viruses often assemble within viral factories, but they sometimes depend on the endoplasmic reticulum (ER), Golgi, and subsequent exocytic pathways for the maturation and folding of their own surface and fusion proteins. This is the case, for instance, for viruses that bud at the plasma membrane such as influenza virus, HIV, and coronaviruses. The henipavirus fusion protein also requires endocytic uptake for cleavage by endosomal proteases, followed by recycling to the cell surface for incorporation into budding viral particles. In this regard, Fischer and colleagues highlight the interdependence of the exocytic and endocytic machinery to produce active viral fusion proteins and infectious Cedar particles, a middle pathogenic henipavirus [17].

The pathways by which viruses leave host cells are complex and difficult to investigate, but it is obvious that they are just as diverse as viral entry pathways, as various exocytic host factors and processes have been observed. Regarding this issue, the laboratory of Urtzi Garaigorta identified Erlin-1, a cholesterol-binding protein in the ER, as a proviral host cell factor participating in the production of infectious hepatitis C virus particles [18]. Chen and colleagues exemplified the central role of ion flow in the trafficking of intracellular vesicles, addressing the importance of calcium channels and pumps in virus penetration, assembly, and exit [19]. The review by Oechslin et al. nicely illustrates all these molecular and cellular processes in the context of hepatitis E virus (HEV), presenting the most recent information on the HEV life cycle, from virus entry and replication to assembly and release of new viral progenies [20].

Viral particles are engaged to transfer the viral genome from an infected cell to a noninfected cell. However, an increasing number of studies on cell-associated viral transmission have indicated the existence of alternative routes for the spread of viral material. Sid Ahmed et al. analyzed how the cellular environment may impact HIV spread in vivo [21]. This review developed from a recent paper by the same group showing that a 3D collagen environment restricts cell-free HIV infection, but promotes cell-associated viral propagation [22]. Other viruses interfere with cell and tissue differentiation as they spread. In this respect, Bilz and colleagues established that rubella virus hampers the formation of embryoid bodies and other 3D cell aggregates derived from human-induced pluripotent stem cells [23]. Whether this blockage is due to viral replication or required for viral spread remains to be clarified. This study suggests that the clinical signs of congenital rubella syndrome are due to an impairment of human development.

In this special issue, I intend to illustrate the possible viral life cycle stages, addressing virology issues from the perspective of cell biology, and from other angles. I strongly encouraged researchers to engage in discussions on the latest knowledge, technical issues, and technological challenges as well as prospects in the fields of viral infections, cell biology, and beyond. Based on 6 reviews, 1 opinion, 12 original research articles, and 1 communication reporting on a wide variety of viruses, this special issue can be considered a success. One of these reviews also demonstrates the interest in using viruses to develop new therapeutic approaches against noninfectious diseases. Nguyen et al. examined the potential of oncolytic viruses genetically modified to encode antitumor interleukine-12 in cancer therapy, with a special focus on herpes simplex virus [24]. Two research articles documenting new methods to study and analyze viral infections include the one by Tekes and colleagues reporting a protocol for culturing organoids derived from the ileum and colon and showing their usefulness in studying feline coronavirus infection [25], and the one by Potratz et al. describing a novel quantitative approach, based on computer analysis of microscopic images to analyze the cell tropism of neurotropic rabies virus [26].

I, however, do not want to give the impression that this special issue provides an exhaustive picture of the cell biology of viral infections. Some discussions are missing, due to a lack of experts in some cases, but the omissions are not deliberate. Virus receptors, endocytic pathways, and further processing of genomic material are important topics. The role of exosomes in virus dissemination and that of protein aggregation in virus-induced diseases are fascinating [27,28]. I also would have liked to provide a greater emphasis on technology, for instance, by including works on nanobodies, electron microscopy techniques, and OMICS. I only hope that this special issue provides the field of viral infection cell biology future research directions and stimulates research in some understudied and nascent areas. Without the fantastic contributions of authors, and the great help of Dolly Wang and other editorial assistants from Cells, this special issue would not have been possible. I would therefore like to thank all these great people.

Lastly, it is difficult in these pandemic times to edit a special issue on the cell biology of viral infections without further mentioning the recent viral zoonotic pandemic and the new coronavirus. In a blueprint list, the World Health Organization (WHO) identified pathogens for which there is an urgent need to develop diagnostics, therapies, and research. The list includes, among others, coronaviruses, and a dozen other zoonotic viruses. The successive epidemics and pandemics of SARS-CoV, Chikungunya virus, Middle East respiratory syndrome coronavirus, EBOV, and Zika virus are all signals over the past 20 years that should have alerted us. Nevertheless, emerging viruses continue to appear of interest only during epidemics and pandemics, which has not contributed to sustained funding, and therefore, to promoting long-term research on emerging zoonotic viruses. Many reasons, too many to discuss herein, are responsible for this situation. We can, however, legitimately speculate about how much time would have been saved in the fight against SARS-CoV version 2.0, if only a few of the financial losses due to global lockdown had been invested in research against emerging viruses, notably coronaviruses. The ten or so viruses on the WHO blueprint list account for less than 5% of the total virology research work referenced by PubMed.gov (Figure 1). Although incomplete, this approach provides a good idea of the collective effort that has been made to date, and as a result, of the important research effort, which remains to be made in the battle against these pathogens and future pandemics.

## Figures and Tables

**Figure 1:**
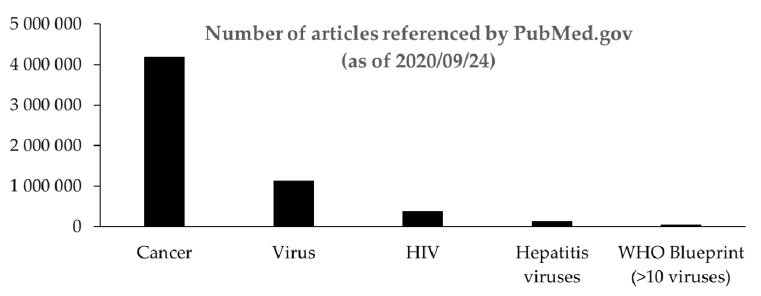
Number of articles on emerging viruses referenced by PuMed.gov. The keywords cancer, virus, HIV, and hepatitis viruses (A to E) were inputted into the search engine. Similarly, the WHO blueprint list represents the sum of results obtained for the next 10 keywords: Crimean–Congo hemorrhagic fever, Ebola, Marburg, Lassa fever, Middle East and severe acute respiratory syndrome coronavirus, Nipah, henipa, Rift Valley fever, and Zika.
